# Climate-driven succession in marine microbiome biodiversity and biogeochemical function

**DOI:** 10.21203/rs.3.rs-4682733/v1

**Published:** 2024-08-16

**Authors:** Alyse A. Larkin, Melissa L. Brock, Adam J. Fagan, Allison R. Moreno, Skylar D. Gerace, Lauren E. Lees, Stacy A. Suarez, Emiley A. Eloe-Fadrosh, Adam Martiny

## Abstract

Seasonal and El Niño-Southern Oscillation (ENSO) warming result in similar ocean changes as predicted with climate change. Climate-driven environmental cycles have strong impacts on microbiome diversity, but impacts on microbiome function are poorly understood. We quantified changes in microbial genomic diversity and functioning over 11 years covering seasonal and ENSO cycles at a coastal site in the southern California Current. We observed seasonal oscillations between large genome lineages during cold, nutrient rich conditions in winter and spring versus small genome lineages, including *Prochlorococcus* and *Pelagibacter* , in summer and fall. Parallel interannual changes separated communities depending on ENSO condition. Biodiversity shifts translated into clear oscillations in microbiome functional potential. Ocean warming induced an ecosystem with less iron but more macronutrient stress genes, depressed organic carbon degradation potential and biomass, and elevated carbon-to-nutrient biomass ratios. The consistent microbial response observed across time-scales points towards large climate-driven changes in marine ecosystems and biogeochemical cycles.

## Introduction

Empirical observations of microbial responses to marine warming are necessary to better understand nonlinear, climate-driven biophysical interactions between the environment and plankton communities. Previous work has demonstrated that warming events, including marine heatwaves, can have profound effects on the biodiversity of microbial communities[1–4]. For example, a 2015-16 marine heatwave in the Tasman Sea resulted in a shift in microbial communities to niche states that resembled locations over 1000 km equatorward[5]. However, these climate-driven impacts on microbial functional potential are poorly quantified. For example, only a limited number of studies have examined metagenomic functional potential for periods longer than 5 years[6–8]. Moreover, differing hypotheses exist as to whether taxonomic changes in microbial communities result in changing ecosystem processes[9–13]. Some studies demonstrate widespread functional redundancy[14,15], while others have observed global variability in functional potential for traits including nutrient uptake[16], predicted growth and replication[17,18], and carbon metabolism[19]. Thus, whether temperature-driven changes in marine microbial communities may impact ecosystem functional potential remains an outstanding question.

El Niño-driven warming events result in many of the same environmental changes as predicted with climate change. However, observations of El Niño's impact on bacterioplankton communities are limited. The 2015 El Niño event was one of the strongest on record in terms of the spatial extent of warming in the Eastern North Pacific[20]. In the California Current, phytoplankton communities shifted to increased cyanobacteria cell abundances[21] and zooplankton communities showed significant changes in composition[22]. In addition, common microbial taxa including SAR11, *Synechococcus*, and *Prochlorococcus* shifted from cold to warm water ecotypes[23,24]. In terms of biogeochemical impact, the 2015 El Niño suppressed primary production[25,26], biomass, and particulate organic matter concentrations, and increased the carbon-to-nutrient ratios[27]. Given the significant impacts on microbial community composition and marine biogeochemistry, the 2015 El Niño offers an opportunity to examine the impact of warming temperatures on microbial metabolism as well as establish links between microbial community composition, functional potential and ecosystem impacts.

Within the California Current Ecosystem, the Southern California Bight (SCB) is a critical transition zone between subpolar and subtropical biomes. ENSO cycles affect the region directly through warming and also indirectly through circulation changes. This region is influenced by the southward, cold, nutrient poor, and low salinity California Current as well as the northward, warm, nutrient rich, and high salinity California Undercurrent[28]. In addition, the SCB is strongly impacted by both point and non-point source pollution, which can induce high levels of localized nutrient loading[29]. Past studies have found clear seasonal cycles in environmental conditions and microbial taxonomic diversity both near- and offshore[27,30,31]. Thus, it represents an excellent model system to test the impact of diverse environmental changes on marine microbiomes.

Here, we combine environmental analyses and metagenomic sequencing from 2011-2022 to quantify links between *in situ* warming, changing nutrient inputs, microbial biodiversity, and ecosystem functions. In particular, we identify the major temporal modes of variation in microbial biodiversity. We hypothesize clear impacts of seasonal and ENSO cycles (including the strong 2015 El Niño event). Thus, we predict that El Niño-driven warming will result in increases of oligotrophic bacterioplankton taxa with a concurrent increase in metabolic strategies associated with oligotrophic communities. We aim to establish a genome-enabled understanding of the climate-driven feedbacks between environmental change, coastal functioning, and biogeochemistry.

## Results

To identify the drivers of seasonal and interannual succession of marine microbial communities, we quantified the environmental conditions using a combination of discrete water samples (nitrate, phosphate, and particulate organic matter) and *in situ* sensors (temperature and chlorophyll). Samples were taken at the MiCRO site located near-shore in the Southern California Bight. In addition, we collected 267 metagenomes covering 11 years (2011–2021) (Supplementary Table 1). These samples spanned all months as well as several stages of the ENSO cycle. A total of 3.47 Tbp were sequenced across all samples.

We observed clear seasonal oscillations in temperature and nutrients (Fig. 1). As usual for this latitude (33˚N), sea-surface temperature peaked between July and September. Nutrients mostly showed an inverse oscillation with high concentrations during the winter and early spring when upwelling occurs[32]. Coinciding with phytoplankton growth (reflected in rising chlorophyll concentrations), there was a nutrient decline between March and June resulting in low concentrations during the summer and fall. However, nitrate and phosphate showed unique trends in the fall, whereby phosphate rose but nitrate stayed at low levels leading to a shift in the dissolved nitrate:phosphate. The ENSO climate cycle also had an imprint on the environmental conditions. Interannually, temperature was depressed during La

Niña conditions from 2011 to 2013, high during the strong El Niño event in 2014–2015, a period of intermediate conditions, and then another La Niña event in 2021. Nutrients generally inversely followed interannual temperature trends with higher levels during La Niña and lower during El Niño conditions. However, phosphate was elevated in 2017 and 2018 despite above average temperature. Thus, we observed a clear correspondence between temperature and nutrients at both seasonal and interannual time-scales but also variation in the relationship between temperature and specific nutrients.

Overall, the microbial community was dominated by a few lineages, and the top 50 families constituted ~ 80% of all sequences (Fig. 2A). The most common putative heterotrophic lineages included *Pelagibacteraceae*, *Rhodobacteraceae*, and *Flavobacteraceae*. The most common photosynthetic families were found within cyanobacteria covering *Synechococcus* and *Prochlorococcus* as well as small eukaryotes like *Mamiellaceae* (incl. *Micromonas*) and *Bathycoccaceae* (incl. *Bathycoccus* and *Ostreococcus*).

These common taxa showed clear seasonal succession (Fig. 2B). In the colder nutrient replete months (Dec to Feb), taxa including *Cytophagaceae*, *Alteromonadaceae*, *Oceanospirillacea*, and *Rhodobiaceae* as well as the photosynthetic *Bathycoccaceae* and ammonia oxidizers *Nitrosopumilaceae* all peaked (see Supplementary Data). In the period with large increases in biomass and falling nutrients (March to May), *Flavobacteraceae*, *Pseudomonadaceae* and *Rhodobacteraceae* showed elevated frequency. During the summer, several non-Proteobacteria families peaked as well as *Synechococcaceae*. In the fall, *Pelagibacteraceae* was common together with the photosynthetic *Prochlorococcaceae* and *Mamiellaceae*. The seasonal cycle in these taxa made a strong community imprint, and community composition clustered by collection month (Fig. 2C). In general, taxa common under cold, nutrient replete conditions have large genomes and vice-versa for taxa during warm, nutrient deplete conditions. In support, we observed a clear oscillation in average genome size with large genomes during the spring bloom period and the smallest at the end of the warm, oligotrophic period, when *Pelagibacteracea* and *Prochlorococcaceae* were common (Extended Data Fig. 2). Hence, linked to the seasonal taxonomic succession was an ecological succession between cells with larger vs. smaller genomes.

Functional gene content demonstrated cycles with near clock-like precision and maximal separation between opposite seasons (Fig. 3). This was consistently seen across different functional classification schemes. Using a multi-table co-inertia analysis (MCOA) of the combined (four) functional classification schemes as well as taxonomic variation showed that the two primary principal components can explain approximately half of the variance (Fig. 3C). Furthermore, the top principal components showed clear annual oscillation (Extended Data Fig. 3). MCOA_PC1 (35% variance) peaked in the fall and thus was slightly offset from the temperature oscillation. Hence, the maximum separation in community composition happened between spring and fall communities. MCOA_PC2 (16%) peaked in the summer and thus followed temperature. Visually, community functional potential clearly separated based on sample month (Fig. 3B and 3C). A comparison across all functional gene profiles reinforced these findings (Fig. 3D and S6). Again, the temporal variation of each functional gene showed strong seasonality. Here, the first principal component (32% variance) separated spring from late fall and the second principal component (11%) separated early summer from winter. Thus, the compositional succession of key taxa coincided with repeated oscillations in microbiome functional genes and overall potential.

Both taxonomic composition and functional potential showed an interannual succession tied to the ENSO climate cycle. The combination of seasonal and interannual shifts captured on average 62% of the total compositional variation (estimated using PERMANOVA, Extended Data Fig. 4). Interannual shifts in temperature correlated with the ENSO cycle (Fig. 4A). During La Niña conditions (negative ONI index), temperature was anomalously low. This was seen in 2011–2013 and then again in 2020–2021. Strong El Niño conditions were present in 2014–2015 and to a lesser extent in 2016–2019. Most microbiome variation was seasonal, but interannual shifts explained an additional 14% of overall frequency variation among all the genes. Interannual variability primarily partitioned into two clusters separating La Niña and El Niño conditions (Extended Data Fig. 5). In support, most genes peaked in frequency either during 2011 (year with the lowest temperature) or 2015 (year with the highest temperature) (Fig. 4B). Nearly half of the interannual variation (46%) was tied to a combination of temperature and nutrient availability (Fig. 4C). Thus, long-term changes in temperature and nutrients made key impacts on this ecosystem. The long-term microbiome changes mimicked the observed seasonal changes. As such, genes most frequent during cold, nutrient replete (La Niña) years had a seasonal peak during the winter months (Fig. 4C). Genes more frequent during warm, nutrient depleted (El Niño) years peaked during the late summer when similar conditions occurred. These functional gene shifts overlapped with biodiversity shifts. Cells with small, streamlined genomes were enriched during El Niño conditions (Extended Data Fig. 2). In contrast, the average genome size peaked during La Niña events including 2012 and 2020. Furthermore, *Prochlorococcacea*, *Synechococceae*, and *Pelagibacteraceae* peaked in the summer and fall but also in years with high temperature and low nutrients (Fig. 4C). In contrast, *Flavobacteraceae*, *Porticoccaceae*, and *Bathycoccaceae* peaked in the spring and were common during colder, nutrient replete conditions. Thus, the same environmental drivers control seasonal and inter-annual cycles of key lineages and functional potential.

We next explored how the seasonal and long-term metagenomic successions affected specific ecosystem and biogeochemical functions. A challenge is the presence of more than 10,000 unique functional genes each with individual annotations. To address this methodological challenge, we trained a 'Natural Language Processing Model' and found that annotations including the term 'iron' were more common in the winter and spring. In contrast, annotations with the words 'nitrate', 'urea', and 'phosphatase' were more common in the summer and fall months. In past analyses of pelagic communities[16], we have detected a distinct biogeography of nutrient acquisition genes indicative of various forms of resource stress in phytoplankton. Seeing this variation in annotation keywords led us to test, if there were similar systematic shifts in biogeochemically important genes in this coastal community dominated by putative heterotrophic bacteria.

First, we confirmed that the frequency of iron acquisition genes were elevated in the winter and spring and peaked in April (Fig. 5A). The maximum frequency of Fe stress genes matched periods with high macronutrient supply in part due to upwelling (Fig. 1). Second, nitrogen and phosphorus stress genes increased in frequency following this period (Fig. 5A). Both gene groups peaked between July and October, when macronutrient concentrations were at a minimum. A taxonomic analysis revealed that these genes were mostly encoded by putative heterotrophs within Proteobacteria and Flavobacteria (Extended Data Figs. 7 and 8). Thus, we observed a clear seasonal succession in putative heterotrophic nutrient acquisition genes linked to the supply of macronutrients.

There was also a parallel change in carbon availability and processing (Fig. 5C). Carbon degradation enzymes (e.g., glycoside hydrolases) were common and constituted 3.6% of all annotated sequences. These genes peaked in frequency in the spring. This seasonal pattern matched the period with high biomass represented by chlorophyll and particulate organic carbon (POC). Furthermore, chlorophyll and POC declined in tandem with nutrient concentrations in the summer and reached a minimum in the fall. This was followed by a similar decline in the frequency of carbon degradation enzymes.

In this ecosystem, plankton constitute a large fraction of the overall particulate organic matter[21]. Thus, we hypothesized that the succession in key microbial biogeochemical functions would make an imprint on the organic matter carbon-to-nutrient ratios, which are commonly high during N or P limitation[33]. In support, we observed that both the carbon-to-nitrogen (C:N) and carbon-to-phosphorus (C:P) ratios were low in the winter and spring when POC was high, macronutrients were high, and N and P acquisition genes were low in frequency (Fig. 5E). In contrast, we observed high C:N and C:P ratios in parallel to the higher frequencies of N and P acquisition genes in the summer and fall. Thus, we detected a clear parallel succession between key nutrient stress markers and the carbon-to-nutrient ratios in organic matter.

As seen for the whole community function potential, we also detected a link between interannual shifts in key functional genes and nutrient and carbon biogeochemistry. The frequency of Fe acquisition genes were elevated during the 2011-13 La Niña years, dropped during the El Niño event in 2014 and 2015, and reached a maximum during the next La Niña in 2021 (Fig. 5B). N acquisition genes were anti-correlated to Fe genes ($r_{spearman}$ = -0.67) and peaked during the El Niño event. P acquisition genes generally followed N genes but reached a maximum in 2018. Carbon degrading enzymes correlated positively with concentration of chlorophyll and POC with maxima during La Niña and a minimum during El Niño events (Fig. 5D). These patterns paralleled shifts in the C:N:P ratios (Fig. 5F). Here, all ratios were low during the La Niña periods and mostly elevated between 2014 and 2019. However, C:N peaked in 2018, whereas C:P and N:P peaked the next year. Hence, the biogeochemical dynamics at the interannual scale was similar to the seasonal scale with colder months matching colder years and vice-versa. This gave rise to a clear ENSO-driven cycle between La Niña years with high macronutrients, Fe stress, and organic carbon processing genes, but low N and P stress genes. The opposite pattern was seen during the El Niño event. Thus, the ENSO cycle led to clear microbiome shifts impacting the biogeochemical coupling between ocean carbon and nutrient cycles.

### Discussion

Our results clearly support a strong link between marine microbiome compositional and functional dynamics at both seasonal and interannual time-scales. One common model posits that while individual taxa are sensitive to environmental change, genetic redundancy leads to resilience in overall microbiome functions[15]. In contrast, common lineages including *Prochlorococcus*, *Synechococcus*, and *Pelagibacter* are all subject to extensive gene gain and losses resulting in connected global phylogenetic and functional biogeographies[34,35]. Furthermore, key lineages display extensive seasonal niche differentiation[6,24]. A time-series from the Mediterranean Sea[12] and now our MiCRO time-series observations suggest parallel taxonomic and functional microbial biodiversity cycles. These observations challenge the notion of functional redundancy in marine microbiomes and instead suggest compositionally as well as functionally very dynamic ecosystems.

Our observations suggested a clear functional succession tied to ocean biogeochemical cycles. The MiCRO site is located in the near-shore environment, where sediment suspension should lead to a high iron flux. Thus, it is unexpected to detect Fe stress under such near-shore conditions, but intermittent iron stress has been seen further off-shore in the California Current ecosystem[36–38]. The local ocean circulation is very dynamic with currents exceeding 0.1 m $s^{-1}$ [39,40], so we are likely observing cells adapted to conditions in the wider region. Spatially, iron stress in connection to elevated vertical nutrient supply, whereas N and P stress are found in warm, highly stratified regions[41,42]. Even regions not commonly regarded as iron-limited like the Iceland Basin can also experience seasonal Fe stress following periods of high nutrient supply[43]. In contrast, macronutrient stress is generally controlled by temperature and stratification and thus peaks in the summer or early fall[44,45]. Our observed seasonal patterns in nutrient stress are consistent with these spatial and temporal dynamics in other systems. ENSO-driven changes to the Equatorial Pacific Ocean revealed a strong positive correlation between temperature and Fe stress[46] and thus the opposite of our observations. However, reduced Fe stress during El Niño events is consistent with stratification and lower macronutrient flux[47]. These observations of nutrient limitation in other regions are derived from phytoplankton dynamics. However, cycles in nutrient stress genes at MiCRO were primarily occurring in putative heterotrophic bacteria. We know less about nutrient limitation in heterotrophic organisms. In another microbiome time-series from the Mediterranean Sea, a spring peak in bacterial Fe stress gene frequencies were also observed[12]. Furthermore, there are reports of Fe stress in heterotrophic organisms in HNLC regions[48] and N and P stress in warm, stratified regions[49]. In the California Current, Fe additions only stimulated bacterial growth when added together with carbon substrates[50]. This suggests some level of regional bacterial iron stress but perhaps not direct Liebig-style limitation. Thus, the microbiome time-series showed functional shifts among mainly heterotrophic microorganisms but matched biogeochemical theory.

Our observations suggest a strong climate sensitivity of marine microbiome biodiversity and functions. Past studies have observed seasonal oscillations in rRNA diversity in many ocean environments[31,51,52]. Both the seasonal and ENSO climate cycles drive oscillations between cold, nutrient rich and warm, nutrient deplete conditions[53]. Such environmental shifts broadly resemble conditions associated with anthropogenically-driven climate change. Specifically, observed tradeoffs between high biomass, Fe-limited conditions and low biomass, N- and P-limited conditions (Fig. 5) are consistent with modeled projections of climate-driven shifts between Fe- and N-limitation and associated impacts on eastern equatorial Pacific ecosystems[54]. Thus, the consistent microbial response observed across both seasonal and interannual time-scales points towards large climate-driven changes in biodiversity, ecosystem functions, and biogeochemistry. For our region, this means an ecosystem increasingly dominated by cells with small genomes including *Prochlorococcus* and *Pelagibacter*, declining organic carbon degradation and POC concentrations, less iron but more macronutrient stress, and cells with elevated carbon-to-nutrient ratios. Such changes will likely have wide impacts on the broader ecosystem.

### Declarations

## Acknowledgments

This work was partially funded by the U.S. Department of Energy Joint Genome Institute Community Science Program 2021. The work (DOI: 10.46936/10.25585/60001365) conducted by the U.S. Department of Energy Joint Genome Institute (https://ror.org/04xm1d337), a DOE Office of Science User Facility, is supported by the Office of Science of the U.S. Department of Energy operated under Contract No. DE-AC02-05CH11231. We also acknowledge support from the National Science Foundation (OCE-2135035 to A.C.M.), National Oceanic and Atmospheric Administration (101813-Z7554214 to A.C.M.), the National Aeronautics and Space Administration (80NSSC21K1654 to A.C.M.), and the National Institutes of Health (T32AI141346 to MLB).

## Data Availability:

Environmental data can be accessed via the MiCRO BCO-DMO data page (DOI:10.26008/1912/bco-dmo.564351.2)[87]. Metagenomic sequencing data is available through the Joint Genome Institute Genome Portal (DOI: 10.46936/10.25585/60001365)[88] and through the National Center for Biotechnology Information Sequence Read Archive (BioProject ID: PRJNA624320). For Supplementary Data, including MiCRO metagenome gene and taxon frequencies and analysis, please see FigShare (DOI: 10.6084/m9.figshare.26082091)[89].

### Methods

#### *Sample collection*

Sample collection occurred at the MiCRO time series at Newport Pier in Newport Beach, California, USA (33.608°N and 117.928°W) between September 2009 and 2021 at daily to monthly (weekly, on average) temporal resolution. Sampling protocols have been previously described[21,23,27]. Four autoclaved bottles were rinsed with nearshore surface water before collection and immediate transport to the lab. A summary of all metagenomic samples is listed in Supplementary Table 1.

A total of 267 DNA samples (replicated) were collected through filtration of 1 L of seawater through a 2.7 µm GF/D and a 0.22 µm polyethersulfone Sterivex filter (Millipore, Darmstadt, Germany) using sterilized tubing and a Masterflex peristaltic pump (Cole-Parmer, Vernon Hills, IL). DNA was preserved with 1,620 µl of lysis buffer (23.4 mg/ml NaCl, 257 mg/ml Sucrose, 50 mM Tris-HCl, 20 mM EDTA) and stored at -20°C before extraction.

Particulate organic matter (POM) was collected using two autoclaved bottles from Newport Pier. Particulate organic carbon/ particulate organic nitrogen (POC/PON) and particulate organic phosphorus (POP) were each collected by filtering 300 ml of seawater through pre-combusted (500°C, 5 hours) 25 mm GF/F filters (Whatman, MA). POC and PON were collected on the same filter. POC/PON and POP both had six replicates collected, a triplicate for each bottle. After filtration, samples were placed on petri-dishes and stored in a -20 °C freezer.

Nutrients were collected from the filtrate of the particulate organic matter (POM) sampling. Sample water initially went through a 25 mm GF/F with a nominal pore size of 0.7 µm (used for POM) and was re-filtered through a 0.2 µm syringe filter into a prewashed 50 ml tube. Filtrate was then stored in a −20°C freezer before the determination of nitrate concentration and phosphate as soluble reactive phosphorus (SRP) concentration.

#### *Shore-station measurements*

Temperature and chlorophyll were recorded via the Southern California Coastal Observing Systems (SCCOOS) automated shore station (Newport Pier) located at the sampling site. Sensors include a Seabird SBE 16plus SeaCAT Conductivity, Temperature, and Pressure recorder and a WetLabs WetSTAR sensor for chlorophyll. Measurements were taken at 4 min resolution, but we transformed the data into daily averages to align with other environmental measurements. The El Niño-Southern Oscillation (ENSO) was measured using the Ocean Nino Index v5 (ONI) from the National Weather Service, Climate Prediction Center.

#### *Nitrate measurements*

From 2011 to 2018: Nitrate samples were thawed in a refrigerator overnight. 50 ml standards were created from an artificial sea water and potassium nitrate solution (10 µM). Standards ranged from 0 µM to 10µM concentration of potassium nitrate solution across 10 vials. 500 µl of ammonium chloride solution (4.7 M) was added to each 50 ml sample and standard and mixed. A sample was poured into a column of copperized cadmium fillings[27,55] and 15 ml was used to flush the system. 25 ml was then collected from the column and 500 µl of sulfanilamide solution was added to the sample, mixed, and allowed to react for 6 minutes. 500 µl of N-(1-Naphthyl)-ethylenediamine dihydrochloride solution was then added to the tube, mixed, and allowed to react for 20 minutes. The sample was read on a spectrophotometer set at a wavelength of 543 nm[55]. From 2018 to 2019, samples were processed using a QuickChem FIA 8500 autoanalyzer (Lachat Instruments, Loveland, Colorado, USA), with a detection limit of 0.014 µmol. Beginning in 2019, samples were processed with spongy cadmium. Spongy cadmium was created with a cadmium sulfate solution (10 g CdSO4 in 50 ml DI water). Zinc sticks are added to the solution and allowed to sit for 8 hours. After sitting, the zinc sticks were washed with 6 N HCl, along with several drops of 6 N HCl added to the CdSO4 solution and drained. Precipitated cadmium was covered with 6 N HCl and stirred to break up the cadmium. The cadmium was drained and rinsed 10 times with DI water. The cadmium in this state was used for this protocol. 5 ml of sample was placed in borosilicate glass tubes (acid-washed) with 1 ml of $NH_4Cl$ solution (0.7 M) added to each. 0.2 g of spongy cadmium was added to each and then capped off, laying horizontally on a mechanical shaker table (100 excursions/ minute) for 90 minutes. 5 ml of sample was pipetted out of the tube and placed in a disposable culture tube. 250 µl of color reagent (mixture 5 g sulfanilamide, 0.5 g N-(1-Naphthyl)-ethylenediamine dihydrochloride mixed in 50 ml phosphoric acid (8.51 M), diluted in 500 ml of nanopure water) was added, vortexed, and allowed to react for 10 minutes. The sample was then read using a spectrophotometer set at a wavelength of 540 nm. Samples were compared to a set of six standard solutions containing artificial sea water and diluted 100 µM $KNO_3$ (standard $KNO_3$ concentration: 0 µM to 60 µM). Standards were treated the same as samples[56].

#### *Phosphate measurements*

SRP concentrations were determined using the magnesium induced co-precipitation (MAGIC) protocol[57,58]. SRP samples to be processed were moved to the refrigerator overnight to thaw. Once thawed, 0.4 ml of 3M NaOH was added to each tube and shaken vigorously. After 5 minutes the samples were placed into a centrifuge set at 1500 g for 20 minutes. Supernatant (P-free seawater) was poured into a separate glass (needed for standards) from the sample tubes and allowed to dry for an hour. 6 ml of 0.25 M HCl was added to all tubes and shaken to dissolve each pellet. 0.66 ml of Arsenate (2:2:1 parts sodium metabisulfite (0.74 M), sodium thiosulfate (88.5 mM), sulfuric acid (3.5 N)) was added to each tube and allowed to react for 15 minutes in the dark. 0.7 ml of mixed reagent (2:5:1:2 parts ammonium molybdate tetrahydrate (24.3 mM), sulfuric acid (5 N), potassium antimonyl tartrate (4.1 mM), and ascorbic acid (0.3 M)) was added to the tubes and allowed to react for 30 minutes in the dark. Samples were then read on a spectrophotometer set to a wavelength of 885 nm, using a 0.125 M HCl solution as black and rinsing agent. Ten standards were made using the P-free seawater collected from the samples and mixed with 1 mM $KH_2PO_4$ creating different dilutions (2 nM to 1 µM $KH_2PO_4$). Standards were treated the same as the samples once made. From 2018 to 2019 samples were sent out to be processed on an auto-analyzer. These samples were also run in parallel with samples using the MAGIC protocol for comparison.

#### *Particulate organic matter*

POC/N samples were processed according to Sharp (1974)[59]. Filters for POC/N were dried at 55 °C for 24 hr and then wrapped with tin discs (CE Elantech). The wrapped filters were combusted in a FlashEA

1112 (Thermo-Scientific) using the NC Soils setup. The oxidation reactor was set to 900 °C, the reduction reactor was set to 680 °C, and the oven was set to 50 °C. Oxygen gas (UN 1072, Airgas) was injected at a flow rate of 250 ml/min, allowing sample combustion to occur at 1,800 °C. Helium gas (UN 1046, Airgas) was used as the carrier gas with the carrier flow rate set to 130 ml/min and the reference flow rate set to 100 ml/min. The compounds serving as standards were acetanilide (71.1% C, 10.4% N) and atropine (70.6% C, 4.8% N). The minimum detection limits for this analysis were 2.4 µg C and 3.0 µg N.

POP was analyzed using a modified ash-hydrolysis protocol[58]. Samples were removed from the freezer and placed in combusted scintillation vials; along with a set of $K_2HPO_4$ (1.0 mM-P) to be used as standards. 2 ml of $MgSO_4$ (0.017 M) was added to each vial, covered in tin foil, and placed in an 80 °C oven overnight. The vials were moved to a 500 °C combustion oven for 2 hours. Once cooled 5 ml of HCl (0.2 M) was added to each vial and put back into the 80 °C oven for 30 minutes. The solution in the vials was transferred to a 15 ml centrifuge tube. Each vial was rinsed with 5 ml of DI water, which was transferred to the respective centrifuge tube. 1 ml of mixed reagent [2:5:1:2 parts ammonium molybdate tetrahydrate (24.3 mM), sulfuric acid (5 N), potassium antimonyl tartrate (4.1 mM), and ascorbic acid (0.3 M)] was added to each centrifuge tube in 30 second intervals and moved to the dark. Tubes were centrifuged for 2 minutes to concentrate any glass fibers or debris to the bottom of the tube. After 30 minutes samples were then analyzed using a spectrophotometer set to a wavelength of 885 nm, with a HCl (0.1 M) solution as a blank and rinsing agent between samples.

#### *Environmental data analysis*

Environmental data was collected at higher sampling frequency compared to DNA. We included all data points to reduce uncertainty for monthly and annual estimates of variation. We fitted a linear model to all data points using 12 monthly and 12 annual (2011 - 2021) categorical factors. This was done in Matlab.

#### *DNA extraction*

To extract DNA, we use an adapted protocol[60]. Sterivex filters were first incubated at 37°C for 30 minutes with lysozyme (50 mg/ml final concentration). Next, Proteinase K (1 mg/ml) and 10% SDS buffer were added and incubated at 55°C overnight. A solution of ice-cold isopropanol (100%) and sodium acetate (245 mg/ml, pH 5.2) was used to precipitate the released DNA, which was then pelleted via centrifuge and resuspended in TE buffer (10 mM Tris-HCl, 1 mM EDTA) in a 37°C water bath for 30 min. DNA was purified using a genomic DNA Clean and Concentrator kit (Zymo Research Corp., Irvine, CA). Finally, extracted DNA was stored at -80°C.

#### *DNA sequencing*

DNA concentration was assessed using a Qubit dsDNA HS assay kit and a Qubit fluorometer (ThermoFisher, Waltham, MA). Next, 30-60 ng of genomic DNA per sample was visually examined via agarose gel electrophoresis to check for degraded DNA. Finally, a Nanodrop ND-1000 (ThermoFisher, Waltham, MA) was used to assess sample purity and verify that A260/A280 values were between 1.6-2.0

and A260/A230 values were between 2.0-2.2. Frozen DNA was transported in 25-500 µl of 1xTE DNA suspension buffer per sample to the DOE Joint Genome Institute (JGI).

A total of 236 samples collected between 2011 and 2020 underwent successful short-read metagenomic sequencing at JGI. After QC, total of 120 samples required additional size selection, thus, an input of 10.0 ng of genomic DNA was sheared around 300 bp using the LE220-Plus Focused-ultrasonicator (Covaris) and size selected with a double SPRI method using Mag-Bind Total Pure NGS beads (Omega Bio-tek). Using the KAPA-HyperPrep kit's (Roche) one-tube chemistry of end-repair, A-tailing, and ligation with NEXTFLEX UDI Barcodes (PerkinElmer), the sample was enriched using 7 cycles of PCR. For all samples, the prepared libraries were quantified using KAPA Biosystems' next-generation sequencing library qPCR kit and run on a Roche LightCycler 480 real-time PCR instrument. Sequencing of the flow cell was performed on the Illumina NovaSeq sequencer using NovaSeq XP V1.5 reagent kits, S4 flowcell, following a 2x151 indexed run recipe. A total of 3.21 Tbp of short-read metagenomic data was produced at an average of 13.6 Gbp/sample, exceeding target sequencing depth.

An additional 31 metagenomic libraries were sequenced from samples collected in 2021-2022 (total 0.259 Tbp, average 8.36 Gbp/sample), this data is available through the National Center for Biotechnology Information Sequence Read Archive (BioProject ID PRJNA624320). For sequence library preparation please see *Supplementary information*.

#### *Assembly and annotation*

Short-read metagenomes were processed following the Joint Genome Institute’s (JGI) metagenomics workflows[61]. Raw paired-end sequences were quality controlled using rqcfilter2 from BBTools (v38.94)[62]. Specifically, ‘bbduk’ was used to remove adapters, perform quality trimming by removing reads where quality drops to zero, removing reads that contain 4 or more “N” bases, removing reads that have an average quality score < 3, and removing reads with a minimum length of < 51 bp. Bbduk was also used for artifact removal of homopolymer stretches of 5 G’s or more at the ends of reads. Bbmap was used to remove reads that matched at 93% identity to host and common microbial contaminant sequences. Read error correction was performed using ‘bbcms’ from BBTools (v38.94) with a minimum count of 2 and a high-count fraction of 0.6. Corrected reads were then assembled using metaSPAdes (v3.15.0)[63] with the ‘metagenome’ flag and kmer sizes of 33, 55, 77, 99, and 127. Contigs smaller than 200 bp were discarded. Assembled reads were mapped back to the contigs to obtain coverage information using ‘bbmap’ from BBTools (v38.94) with ‘interleaved’ as true, ‘ambiguous’ as random, and the ‘covstats’ option specifying a contig coverage file. The assembled reads were structurally annotated using tRNAscan-SE (v2.0)[64], RFAM[65], CRT-CLI (v1.8), Prodigal (v2.6.3)[66], and GeneMarkS-2 (v1.07)[67] as previously described[61]. Structural annotation results were merged to create a consensus structural annotation, which was then used for functional annotation. On average, 71.99% of quality filtered reads (Minimum: 22.67%, Maximum: 100%) were assembled.

Functional annotations were predicted using Last (v983)[68] and custom hidden Markov models implemented through HMMER (v3.1b2)[69] and TMHMM (v2.0)[70]. Functional annotations were assigned using multiple protein family databases: KO[71], EC[72], COG[73], TIGRFAM[74], and Pfam[75]. Phylogenetic assignments for each read were assigned based on the best Last hits of the protein coding genes (CDSs). A consensus phylogenetic assignment for each contig was generated using a majority rule, whereby the lineage at the lowest taxonomic rank to which at least 50% of CDSs on the contig was assigned. JGI's pipeline can be implemented using the National Microbiome Data Collaborative's (NMDC) open-source online platform Empowering the Development of Genomics Expertise (EDGE)[76].

#### *Community analysis*

The microbiome time-series analysis was based on many of the recommendations from Coenen et al (2020)[77]. The unit of biodiversity (akin to an OTU) is either taxonomic (at 'Family' level) or unique protein families using primarily KEGG Orthologs (KO) but also COG, Pfam, or TIGRFAM classifications. Occurrence was normalized to summed total coverage for each sample. We excluded biodiversity units with a frequency below 5E-5 resulting in a low occurrence of absent units (i.e., zeros). Only having a few instances of zeros for the relative abundances allowed us to use linear models (e.g., PCA) rather than applying non-linear tools (like Bray-Curtis similarity and PCoA) when comparing samples or units of biodiversity.

We normalized each biodiversity unit to a mean of zero and a standard deviation of one (i.e., z-score) when analyzing the temporal variation of individual taxa or genes. From this, we fitted models with monthly (12 levels representing seasonal variation) and yearly (12 levels representing interannual variation) categorical factors. A low frequency band-pass filter using 'lowess' (Matlab 'smoothdata' function) was applied when assessing temporal changes. There was a strong autocorrelation with temporally adjacent samples sharing 92.5% taxonomic and 98.7% functional similarity.

#### *Average genome size*

The average genome size was calculated according to the standard JGI IMG pipeline. It was estimated as: $N_{Bases\_assembly}$ / $N_{Genomes}$. $N_{Genomes}$, the median coverage of conserved single-copy core genes (139 marker genes with associated Pfam IDs) as described previously[78].

#### *Compositional variance analysis*

We used PERMANOVA[79,80] to quantify whole community composition variance associated with seasonal and interannual changes. We used sampling month and year as well as their interactions as factors. Community composition was estimated using taxonomic (family level) or functional (COG, KO, Pfam, and TIGRfam) changes. We estimated the Euclidean distance of the normalized frequency of each unit of biodiversity for the compositional matrix. We used 'adonis' from the 'vegan' package (v2.6-4) in R[81,82] for this analysis.

#### *Multi-table co-inertia analysis*

A joint principal component analysis of both taxonomic (family level) and functional changes (COG, KO, PFam, and TIGRFam) was performed as previously described[83]. In brief, we used Multi-table Co-Intertia Analysis from the 'ade4' package (v1.7-22) in R[84,85]. The first two principal components are presented from this analysis.

#### *Annotation keyword model*

A 'Natural Language Processing Model' was trained on all gene annotations. This was done using the 'spaCy' Python package[86]. Subsequently, we calculated the time-series of all keywords weighted by the relative frequency of the associated gene. Next, we found the seasonal oscillation and annual change of each keyword.

## Figures

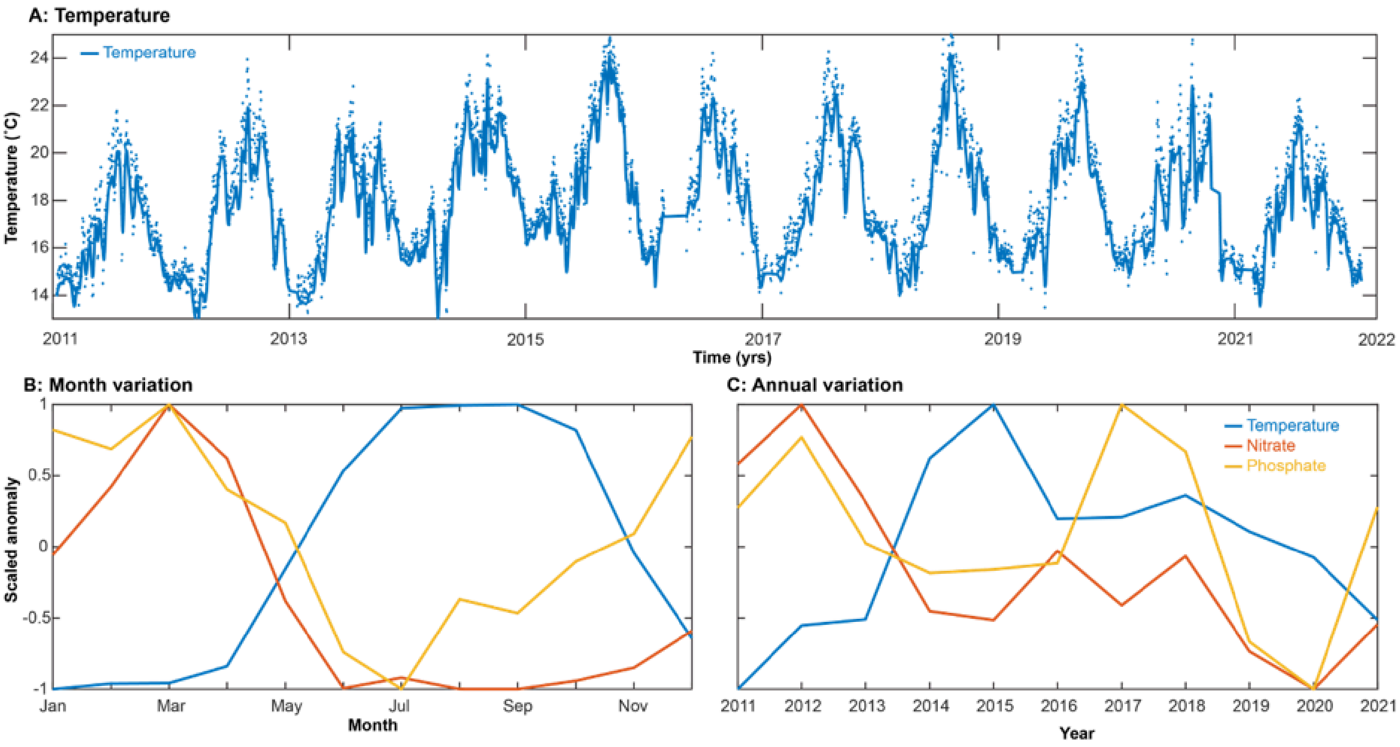

### Figure 1

**Environmental dynamics across multi time-scales. A:** Temperature (˚C) measured at the SCCOOS automated shore-station, **B:** Monthly anomaly in temperature, nitrate and phosphate. **C:**2011 to 2021 annual anomalies in temperature, nitrate and phosphate. The anomalies were scaled from -1 to 1. The full time-series of nitrate and phosphate are presented in Extended Data Fig. 1.

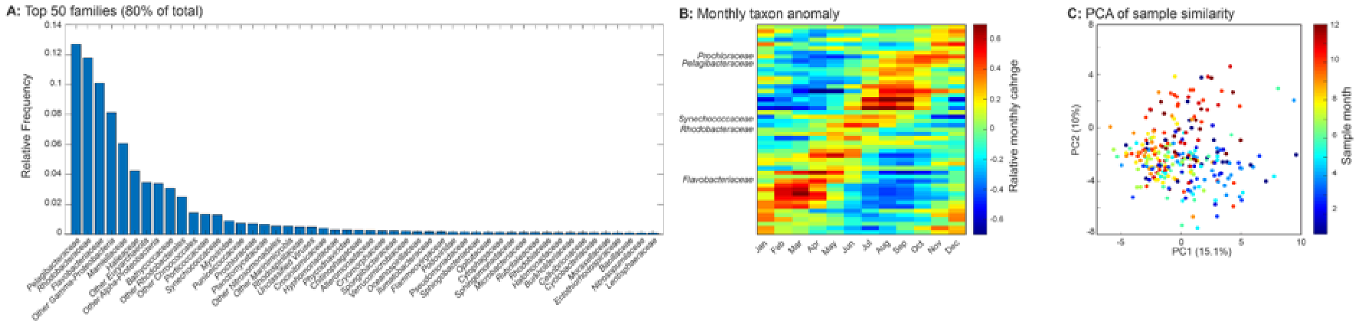

### Figure 2

**Dominance and seasonal dynamics of key lineages. A:** Rank abundance of top 50 lineages. The top 50 lineages (defined at the family level) represented 80% of all lineages detected (n = 929). **B:** Seasonal succession of top 50 lineages. A linear model with monthly and yearly factors were fitted to the z-score normalized relative abundance of each lineage. Here, monthly factors represent the difference in frequency associated with that time-frame. The lineages were ordered by peak month. Key functional types are labeled. **C:**Principal component analysis (PCA) of the z-score normalized relative abundance of the top 50 families within each sample (n=267). Each sample is colored by collection month to illustrate the importance of seasonality for community composition.

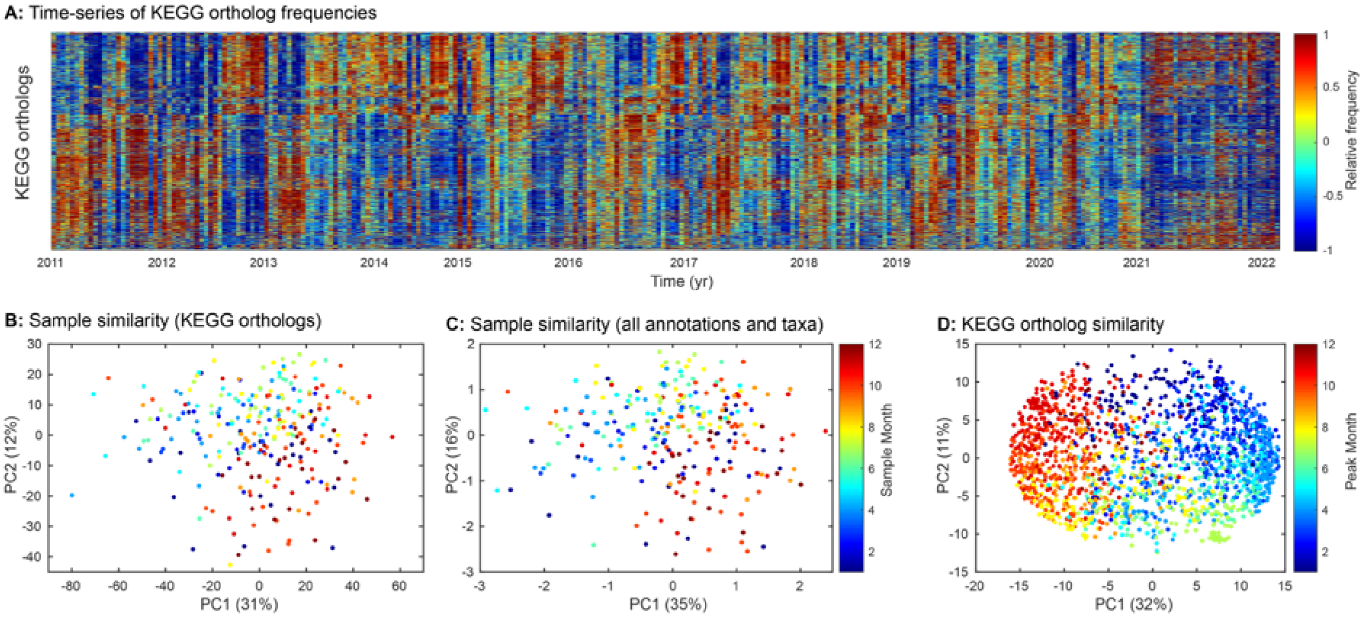

### Figure 3

**Seasonal and long-term succession in microbial community functional potential. A:** Time-series of functional genes annotated with the KEGG ortholog system. Time-series using other annotation systems (COG, PFam, and TIGRfam) show similar temporal patterns. The frequency of each gene was Z-score normalized across time. **B:** Temporal dynamics of community functional potential. Sample similarity was estimated from Z-score normalized relative abundances of each KEGG ortholog using PCA. **C:** Integrated community functional similarity using multi-table co-inertia analysis (MCOA). This integrated analysis used annotations from KEGG, COG, PFam, and TIGRfam as well as taxonomic variation. **D:** Similarity in temporal dynamics of KEGG orthologs. The input data is the same as in Fig. 3B but here we quantified the temporal dynamics of functional genes (rather than samples) using PCA. Each gene is colored by peak month illustrating the importance of seasonal dynamics in structuring the variation in gene frequency. The time-series for COG, PFam, and TIGRfam are presented in Extended Data Fig. 6.

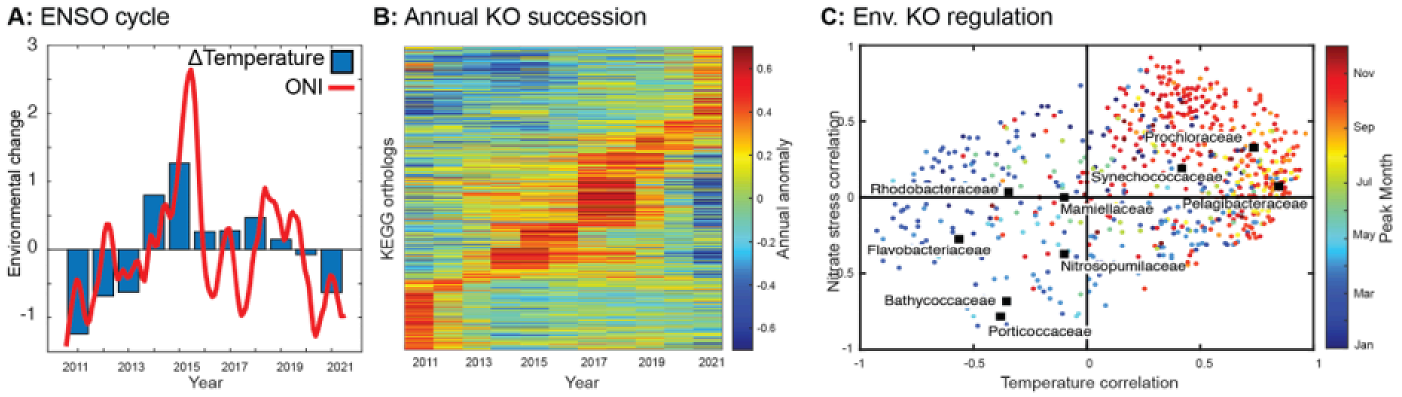

### Figure 4

**Interannual changes in microbiome taxa and functions. A:**Link between ENSO phases (ONI, red line) and interannual temperature anomalies (blue bars) at MiCRO. **B:** Interannual succession in microbiome functions. Interannual anomalies for each are sorted according to peak positive year. **C:**Spearman correlation between interannual changes in temperature and nitrate stress (-1 * [nitrate]) vs. interannual changes in normalized taxa or gene frequencies. Here, we show the interannual changes using KEGG annotations but similar patterns are seen using other annotation systems.

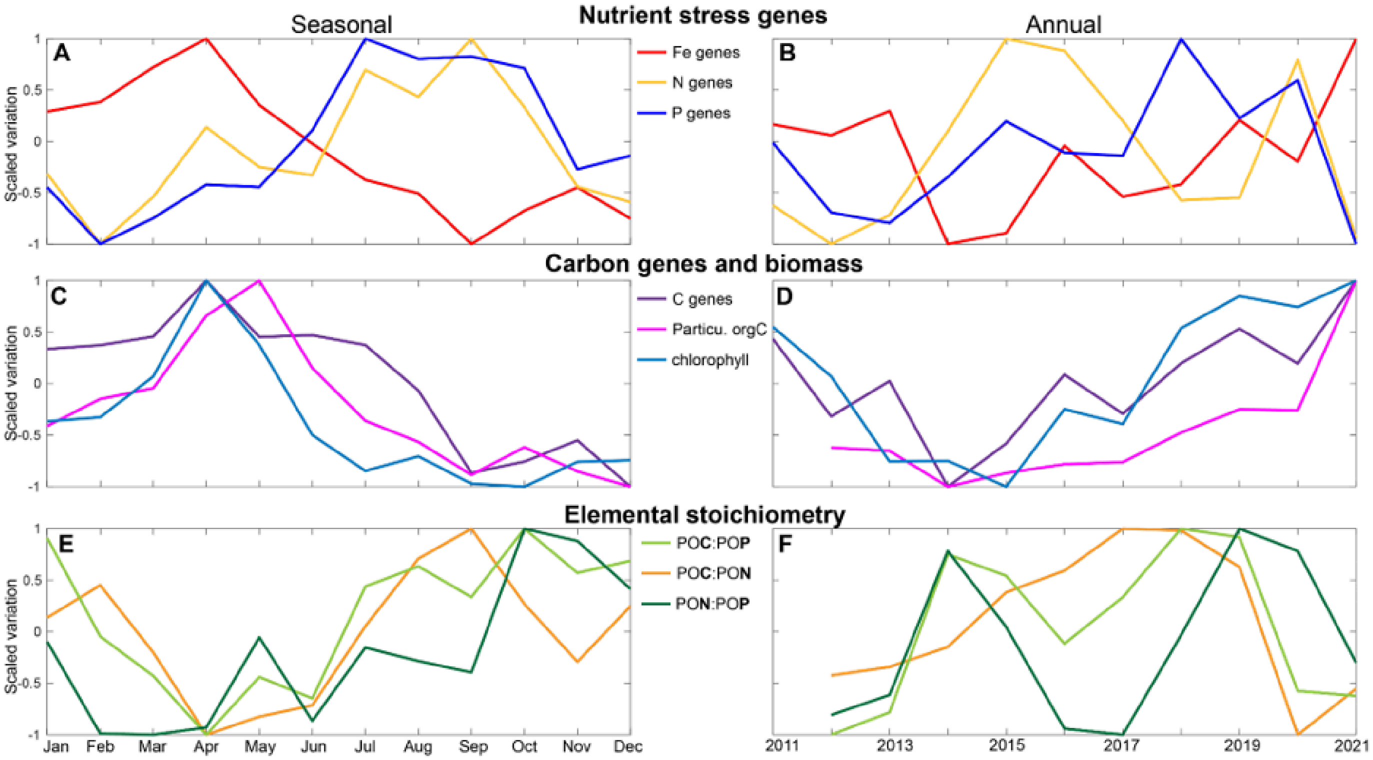

#### Figure 5

**Biogeochemical succession at seasonal and interannual time-scale.** Scale seasonal anomaly for (A) nutrient cycling genes, (C) carbon processing genes, particulate organic carbon (POC), and chlorophyll, and (E) elemental stoichiometry including C:P, C:N, and N:P. Scaled interannual anomaly for (B) nutrient cycling genes, (D) CAZymes (carbohydrate degrading enzymes), particulate organic carbon (POC), and chlorophyll, and (F) elemental stoichiometry including C:P, C:N, and N:P. Seasonal and interannual anomalies are scaled from -1 to 1. See Supplementary Table 2 for a list of genes linked to each biogeochemical category. See Extended Data Fig. 7 and 8 for the individual variation in each gene and a summary of phylogenetic affiliation.

## Supplementary Files

This is a list of supplementary files associated with this preprint. Click to download.

- SupplementaryTable1Larkin2024.csv
- SupplementaryTable2Larkin2024.csv
- LarkinSupplementaryNatureMicrobio2024.docx
